# The experience of mental health service users in health system strengthening: lessons from Uganda

**DOI:** 10.1186/s13033-019-0316-5

**Published:** 2019-09-06

**Authors:** James Mugisha, Charlotte Hanlon, Birthe Loa Knizek, Joshua Ssebunnya, Davy Vancampfort, Eugene Kinyanda, Fred Kigozi

**Affiliations:** 1 Kyambogo University, Kampala, Uganda; 2 Butabika National Referral and Teaching Mental Hospital, Kampala, Uganda; 3 Centre for Global Mental Health, Health Service and Population Research Department, Institute of Psychiatry, Psychology and Neuroscience, King’s College London, London, UK; 4 Department of Psychiatry, College of Health Sciences, School of Medicine, Addis Ababa University, Addis Ababa, Ethiopia; 5 Faculty of Medicine and Health Sciences, Norwegian University of Science and Technology, Trondheim, Norway; 6 KU Leuven Department of Rehabilitation Sciences, Leuven, Belgium; 7 University Psychiatric Centre KU Leuven, Kortenberg, Belgium; 8 MRC/UVRI Uganda Research Unit on AIDS, Entebbe, Uganda; 9 Department of Psychiatry, Makerere University, College of Health Sciences, Kampala, Uganda; 10 PO. Box 2958, Kampala, Uganda

**Keywords:** Service user involvement, Mental health systems strengthening, Uganda

## Abstract

**Background:**

Mental, neurological and substance use disorders are a public health burden in Uganda. Mental health service user involvement could be an important strategy for advocacy and improving service delivery, particularly as Uganda redoubles its efforts to integrate mental health into primary health care (PHC). However, little is known on the most effective way to involve service users in mental health system strengthening.

**Methods:**

This was a qualitative key informant interview study. At national level, 4 interviews were conducted with national level health workers and 3 service user organization representatives. At the district level, 2 interviews were conducted with district level health workers and 5 service user organization representatives. Data were analyzed using content thematic analysis.

**Findings:**

Overall, there was low mental service user participation in health system strengthening at both national and district levels. Health system strengthening activities included policy development, implementation of programs and research. Informants mentioned several barriers to service user involvement in mental health system strengthening. These were grouped into three categories: institutional, community and individual level factors. Institutional level barriers included: limited funding to form, train and develop mental health service user groups, institutional stigma and patronage by founder members of user organizations. Community level barriers included: abject poverty and community stigma. Individual level barriers included: low levels of awareness and presence of self-stigma. Informants also recommended some strategies to enhance service user involvement.

**Conclusion:**

The Uganda Ministry of Health should develop a strategy to improve service user participation in mental health system strengthening. This requires an appreciation of the importance of service users in improving service delivery. To address the barriers to service user involvement identified in this study requires concerted efforts by the Uganda Ministry of Health and the district health services, specifically with regard to attitudes of health workers, dealing with stigma at all levels, raising awareness about the rights of service users to participate in health systems strengthening activities, building capacity and financial empowerment of service user organizations.

## Background

There is a growing interest in the involvement of service users in health systems development [1–5], although this is still a recent innovation in many of the service delivery systems around the world [6]. Collaboration with users provides key inputs into the health system to deliver integrated and quality services that meet the needs of the populations they are designed to serve [7–9]. For example, service users can share their lived experience (e.g., with stigma, service seeking and the attitudes of service providers towards people with mental illness) and this information can be used to improve health reforms and service delivery [6, 10]. Evidence relating to collaboration with service users is more available from high-income as opposed to low-income countries [10–13]. The structure(s) and benefits of service user involvement are, therefore, more evident in high-income countries such as Canada, the United States, Great Britain and Australia where health reforms have been undertaken to support the involvement of service users [5]. As a result, health systems have been reformed to the benefit of the users [6].

Health systems in Africa are weak and there is a pressing need for effective strategies to improve their response and functionality [14, 15]. Collaborating with service users is one of the strategies that can be adopted to achieve this objective [1–4, 10]. The majority of the people in Africa with mental disorders or psychological problems do not receive any effective treatment or care [2, 16]. Of late, the public health burden of mental health problems is being recognized and mental health has gained some level of significance in some countries on the continent [14, 17]. It has been reported that between 2000 and 2015, the continent’s population grew by 49%, and the number of years lost to disability as a result of mental and substance use disorders increased by 52% [15]. It is also reported that in 2015, 17.9 million years were lost to disability as a consequence of mental health problems in the region [17]. Africa’s population is expected to double over the next three decades and this turns mental health into a priority sector on the continent [17]. In view of these challenges, Africa adopted a new Mental Health Policy Framework (MHPF) and Strategic Plan 2013–2020 [14, 18, 19]. The MHPF and Strategic Plan (2013–2020) was developed as one of the ways to operationalize the World Health Organization’s Mental Health Action Plan (2013–2020) [14]. Integration of mental health into primary health care (PHC) is one of the strategies highlighted in this framework to improve access and care for people with mental illness. This framework (MHPF) focuses on vulnerable people and community based agencies fostering participation of service users/vulnerable people in service delivery [20].

Service user involvement an available and vital strategy that can be used to increase access and effectiveness of mental health services in Africa. Service users can be involved in participatory planning, monitoring and evaluation to improve the quality and appropriateness of care [1–4, 10]. Some studies have pointed to enormous human resource gaps in Africa [14, 15]. This human resource gap can be addressed by training service users in aspects of mental health care delivery [1]. Furthermore, given that stigma associated with mental illness is still a reality on the African continent [21, 22], service users can contribute by mobilizing families and communities on issues related to mental health stigma [3, 4].

In Uganda, mental, neurological and substance use disorders (NMS) are a major public health burden. For example, due to the two decades of civil conflict in northern Uganda, the region had one of the highest levels of post-traumatic stress disorder in the world, with an estimated prevalence of 54% [23]. Relatedly, our studies from 7 years post-conflict still indicate a high burden of mental disorders with 54% reporting symptoms of PTSD, and 67% reporting depressive symptoms [23]. Risk factors for mental health problems are widespread. Beyond the civil conflict, there is also a high burden of HIV/AIDS, increasing urban and rural poverty, and urbanization which contribute to the high public health burden of mental, neurological and substance use disorders in Uganda [23–25].

Despite this high burden, the health sector is poorly funded. The overall responsibility to deliver mental health services in Uganda belongs to the Ministry of Health [26, 27]. Uganda spends only USD 33 per capita on health, which is far below the regional average (Uganda Ministry of Health, 2011) [28] and the situation has not changed much today. Moreover, in contrast to other countries in the region, public financing of health in Uganda is low at 22.6% of total health expenditures and there is consensus that the health sector is underfinanced and cannot deliver the Uganda National Minimum Health Care Package (UNMHCP) [27, 29]. Donor funding of the health sector is more than 32% and out of pocket spending is more than 54% of the health expenditure [29]. Mental health attracts less than 4% of the entire health sector budget [28, 30]. The country also depends on obsolete laws and policies, which cannot guarantee enforcement of rights or help in seeking redress by those with mental illness [15]. The mental health policy was approved in 2011, after being in draft form for over 11 years, but is still not widely disseminated to the general public and key service sectors [15, 28].

Collaboration with service users could help to address some of these challenges. Some user movements have started to evolve in Uganda at both national and district levels. The views of service users on health systems strengthening are deemed vital in this study as the country continues to strengthen its plans to scale-up access to mental health care through integration into PHC.

This study was carried out as part of the multi-country EMERALD project (Emerging Mental Health Systems in Low- and Middle-Income Countries) [31]. EMERALD covered four countries in Africa (Ethiopia, Nigeria, South Africa and Uganda) and two countries in Asia (India and Nepal). The project was supported by five different European countries (UK, The Netherlands, Spain, Switzerland and France) [32]. The aim of Emerald was to identify key health system barriers to, and to provide evidence-based solutions for, the scale-up of mental health services in LMICs [32]. The longer-term aim was to improve mental healthcare, and so contribute to a reduction in the large treatment gap for mental disorders [33]. A key component of Emerald was to strengthen service user involvement in mental health system strengthening. A cross-country study was conducted with the aim to explore barriers to, and facilitators of, service user/caregiver involvement in mental health systems strengthening. Findings from the cross-country analysis and the individual country analyses in Ethiopia [34] India [35] and Nepal [3] have been published to date. In this paper, we present the Ugandan findings on the experience of mental health service users in involvement in policy development, service planning, research, and service monitoring aspects of health system strengthening.

To this end, we will use the socio-ecological model [36]. The socio-ecological model is an appealing conceptual tool for guiding public health interventions and research [37, 38]. It recognizes the dynamic interaction among personal and environmental factors, including family, school, community and mental health agencies [38]. As noted, essential findings from research using the socio-ecological model have been used for health policy reform and service development [38]. In this study, we presuppose that services users’ abilities to engage in the health system (strengthening) are not only shaped by their abilities, attitudes, beliefs and values (and other personal characteristics such as gender, age, religious identity, racial/ethnic identity, sexual orientation, economic status) but also by community (both formal and informal networks and social support systems), organizational (e.g., rules and regulation) and policy/enabling (global and local policies) factors in the larger society milieu [39].

## Methods

### Study design

We undertook an exploratory qualitative study. The study is regarded as exploratory because it is the first study in Uganda to focus on mental health service user experience with health systems strengthening. Qualitative methods were employed because they are deemed appropriate in exploratory studies [40, 41]. Qualitative methods are also useful in studying context [41, 42]. The value of semi-structured interviews has been well documented [41, 43, 44]. Semi-structured interviews allow a substantial amount of control over the interview session by the informant and they can meaningfully collect a large amount of data that can deepen the understanding of the phenomenon being studied [43, 44].

### Study population

This study was conducted in Uganda. The country lies in East Africa and across the equator, about 800 km inland from the Indian Ocean [45]. Uganda is a landlocked country, bordering with Kenya in the East; South Sudan in the North; Democratic Republic of Congo in the West; Tanzania in the South; and Rwanda towards the South West [45]. The country is divided into districts, which are further subdivided into Counties, Sub-counties and Parishes. The total population of Uganda was 34.6 million persons in 2014 [45].

At the national level, our study was carried out in Kampala, the capital city of Uganda with a population around 1,507,080 people [42]. The area is relatively well serviced with social services. It is home to the two national referral hospitals for mental health care, i.e. Mulago National Referral and Teaching Hospital and Butabika National Referral and Teaching Hospital. Most of the population is urban with a few rural confines. The informants interviewed at the national level worked at the national referral hospital, Butabika National Referral and Teaching Mental Hospital and national level non-government organizations (NGOs). The national level hospitals in Uganda are the highest level of service delivery and provide specialized services in addition to some general health care [29]. The national level NGOs normally operate countrywide and normally have more resources compared to the district level NGOs.

Our study population at the district level was drawn from Kamuli district. Kamuli district is located in Eastern Uganda. The district was purposively selected. It is part of the Programme for Improving Mental Health Care (PRIME) [46]. Kamuli was selected as a PRIME study site because of its rural nature and limited access to health services. The Programme for Mental Health Care research consortium aimed to implement and evaluate mental health care plans (MHCPs) for adults in five LMIC districts as part of generating high quality evidence on integrating mental health into primary care in low resource settings. The plan addressed selected priority mental disorders including major depression, alcohol use disorders, psychosis and epilepsy (which are treated within mental health care services in Uganda). In partnership with the Ministry of Health, Uganda and using the World Health Organization Mental Health Gap Action Programme (mhGAP) Intervention Guide [47], the PRIME Uganda study team developed, implemented and evaluated the MHCP for Kamuli district. The process of developing the plan was further informed by the Theory of Change Framework [48] with involvement of several stakeholders, including PHC workers, health managers, political leaders, service users and lay opinion leaders from the district. Implementation was at three levels of the district health system namely: health care organization, health facility and community level. Five components of care were developed and these included knowledge enhancement, detection, treatment, follow-up and health management. As part of the implementation and in order to evaluate the MHCP, all the primary health care workers (nurses, midwives and clinical officers) in the thirteen health facilities, including a number of selected community health workers of Kamuli District, were trained in mental health care/intervention using the adapted WHO mhGAP Intervention Guide. Details of the Kamuli district MHCP have been described elsewhere [49]. The district is largely rural with a few semi-urban confines and with an estimated population of about 500,800. The majority of the population are peasant farmers growing mainly sugar cane, maize, beans and groundnuts on a small scale. Health care in this district is provided at the district, sub-county, parish and community level.

### Sample selection

This was done at two levels, the national level and district level.

#### National level interviews

National level interviews were conducted with mental health specialists/policy makers at Butabika National Referral and Teaching Hospital (n = 4). We selected informants from Butabika because it is the leading hospital in the country in providing mental health care and it has a long history in working with service users. Service user informants (n = 3) at this level were selected mainly because of their direct experience engaging with policy makers at national level, but also their direct experience of using services from specialized units at Butabika Hospital. With this experience, they could provide unique insights into what works (in the social service delivery system), and their experiences of stigma and discrimination, livelihood challenges and the economic constraints faced by users. These insights are likely to shade some light on the overall quality of services and governance challenges faced by the delivery system [4, 6].

#### District level interviews

At the district level, interviews were conducted with health service managers and policy makers (n = 2) because of their direct involvement with user organizations at this level. Service user experience with mental health strengthening activities was captured by selecting informants from user organizations (n = 5) at this level. Their background enabled them to provide useful insights on stigma, discrimination, the law on mental health and livelihood challenges [3, 47].

The socio-demographic characteristics of the sample are summarized in Tables 1 and 2.

**Table 1:** Characteristics of study informants

Informants	Number
National level	
Senior psychiatrists	3
Senior psychologist	1
Service user organizations	3
District level	
Policy makers	2
Service user organizations	5

**Table 2:** Socio-demographic characteristics of informants

Characteristics	Senior health workers/policy makers National level	Senior health workers/policy makers District level	Service users National level	Service users District level
Age (years; mean and range)	4 (40–68)	2 (40–50)	3 (38–50)	28–45
Gender				
Male	4	2	1	3
Female	0	1	2	2
Median education level	Graduate	Graduate	Diploma	Primary level

### Study instrument

We used a key informant topic guide which was adapted from the cross-country Emerald topic guide to fit the Uganda context. The interview guide covered, service user involvement in policy making, mental health research and evaluation of mental health services. Our participants shared their experience with (i) mental research, (ii) their role in health service strengthening, (iii) barriers in health service strengthening, and (iv) recommendations relating to user involvement in health service strengthening. We also used our experience as senior mental health workers to enrich the instruments. The first three interviews were piloted. The feedback from this process was used to improve the process of data collection and quality of the guide.

National level interviews at the hospital were collected through arranging appointments with the Hospital Executive Director. The interviews with health workers were conducted in their offices during working hours. National level interviews with service users carried out at Butabika hospital at the project office. Participants were approached through Chief Executives of their agencies and we did not witness any refusals. All interviews at national level were conducted in English.

At the district level, the health workers/policy makers that participated in this study were approached through the District Health Officers (the heads of health services at district level). Appointments were arranged through the District Health Office and selected health workers were interviewed in their offices. All interviews with health workers at this level were conducted in English. Service users selected for interview at this level were selected through the leaders of their organization and appointments were arranged at the community level. Interviews at the district level with service users were conducted in the local language (Lusoga) and translated into English.

All interviews were audio recorded and lasted between 45 min and one hour. The interviews were conducted by the first author, an experienced mental health researcher, with help of a research assistant. Informants were given adequate space and time to ask any questions that related to the study.

### Data analysis

Content thematic analysis was the main method of data analysis. Content thematic analysis involves breaking the text into small units of content and submitting them to descriptive treatments [49]. In doing content thematic analysis, we were guided by frameworks by Braun and Clarke et al. [49]. And, because we were working within an ecological framework, we developed categories we thought could influence participation in health systems strengthening, including policy context, community and individual factors. All the initial coding was undertaken by the first author to develop tentative codes. The tentative codes were shared and improved by all members of the research group. Under each category, we developed themes and later subthemes emerged, which were compared systematically across both national level and district level interviews. The emerging themes and subthemes were shared out with the research team and agreed upon throughout the entire process of data analysis. In the process of conducting and analyzing the interviews, we were aware of our position as health workers and how this could influence the results. Consequently, emerging themes and subthemes were shared with the research teams so as to develop a common interpretation.

### Ethical considerations

Ethical approval was secured from Mengo Research Review Committee (MHRRC) at Mengo Hospital, Uganda. Approval was also secured from the Butabika hospital internal research review committee. All informants were briefed about the objectives of the study and provided informed consent. Consent forms were signed with health care professionals/policy makers in the study while members of the service user organizations gave verbal consent due to their limited level of education. Informants were assured of confidentiality and all data collected were kept securely and were only accessible to the research group. Informants were compensated for their participation in this study as guided by ethical boards in Uganda and as provided for by the Uganda National Council for Science and Technology.

## Results

In this section, we present the experience and level of service user involvement in mental health system strengthening both at national and district levels as well as the common barriers to service user involvement. The latter are grouped into three categories: institutional, community and individual level barriers.

### Experience of services users in mental health systems development

We asked our informants both at national and district levels about their experience with mental health service development and research. Our findings indicated that at the national level, there was a low level of mental health service user involvement in health system strengthening. It was reported that mental health service users had not been involved in policy development activities:

*“Of course not, we were not consulted at all as users…. you read about them [policies and legislations] in the newspapers … but honestly being brought on board and being asked what your opinion is, no one can ask [you for your opinion]” (National Level Informant, Service User Organization A).*

In a similar vein, another informant affirmed:

*“…we just see things written [in policy documents], yeah when you Google, you read about them, but not even somebody giving you a phone call and say let’s have a meeting somewhere… no, nobody has asked” (National Level Informant Service User Organization B).*

Informants argued that policy makers at the Uganda Ministry of Health preferred to be more paternalistic rather than service-user centered in policy development. Given their high professional hierarchy on mental health issues, they preferred to act on behalf of service users:

*“They [policy makers] assume that they know the plight and experience of mental health service users and therefore take on the duty to represent us (Informant National Level, Service User Organization B).*

*“Doctors like you [points to first author] always look at us as sick and therefore not competent to contribute to matters that affect us” (Informant National Level, Service User Organization A).*

*“…the doctors….of a certain age [senior doctors] feel they know everything [that concerns a patient]” (Informant National Level, Service User Organization A).*

It was noted that in most cases, mental health service users were only consulted if it was a donor requirement for funding a program. Even then, they were normally engaged through national level umbrella organizations (these coordinate several activities on behalf of other organizations, such as the National Union of Disabled Persons in Uganda, NUDIPU).

*Sometimes we come in under NUDIPU; which seems to be a national level entity and some people have the thinking that this is where we belong (Informant National Level, Service User Organization A).*

The participating mental health service users however strongly resented this arrangement of consulting them through umbrella organizations. Their perspective was that NUDIPU was formed by those who had physical disability and therefore could not adequately champion issues for those with mental health problems because they, in most cases, have no physical disability.

The informants argued that fair or adequate representation was compromised when the leaders of umbrella organizations invite only a few users (and at worst none) during policy consultative meetings. This kept them in the shadows of policy making.

*“…representation here is not fair [in umbrella organizations]…am telling the truth… sometimes you will be alone …, so your voice is not heard, we are hardly there and…almost invisible” (National Level Informant, Service User Organization, B).*

In contrast, the policy makers interviewed at national level did not perceive any unfair representation of mental health service users. They seemed comfortable with not including service users with the justification that policy development was a technical matter beyond the comprehension and capacity of service users.

Furthermore, policy-makers mentioned that it would be very expensive to reach out fairly to all service user groups in the country.

*Ministries are always stuck in terms of resource envelopes and no one can afford to invite all the users within a national framework. It is just not cost effective (National Level Mental Health Worker/Policy Maker III)*

Furthermore, there was limited involvement in policy development at the district level within the Uganda Ministry of Health structures and no service user or service user organization reported having participated in policy development activities. This was partly attributed to the fact that organizations were considered to be young (less than 3 years old) without systematic activities, like those at national level.

However, some informants expressed optimism that the Uganda Ministry of Health, with support from World Vision Uganda (a non-governmental organization), had trained some service user organizations, which were sufficiently empowered and could engage in policy related activities.

Furthermore, there were no reports of involving mental health service users in planning for mental health services at district level due to limited budgets and low prioritization of the mental health at district level.

Overall, the understanding of most informants was that policy development is a more abstract topic. Many of the members of the user groups did not have a clear understanding of policy neither did they find it important to take part in policy development. Service users identified a number of benefits of participating in health systems strengthening, including stigma reduction, improving the quality of services and the potential of closing some of the human resource gaps in the health system through task shifting.

### User involvement in mental health research

As with policy development, at national level, service user involvement in mental health research was limited. Only a few of our informants mentioned hospital-based research where occasionally a few service users are involved. Informants further noted that other hospitals at a lower level (regional and district hospitals) rarely have research projects on mental health. The few mental health service users that were involved in mental health research described the process as largely extractive as they were never given feedback and were of the view that their input was not valued.

*“One gets into the feeling that they just want to satisfy a process but not that they are really interested in our views. That is why they will never give us feedback” (National Level Informant, Service User Organization I).*

At the district level, many of the informants were not sure whether research was vital in health systems development and whether it was necessary for them to participate in the research process.

### Service monitoring

Nearly all informants at national level reported that the Uganda Ministry of Health undertakes monitoring of mental health services, although service users are hardly involved in any way. They affirmed that users were only involved in monitoring when it was a donor condition or loosely as part of a research project (though this was also rare).

At the district level, users were struggling to grasp the concept of monitoring and had little idea as to how they could be involved in monitoring.

### Barriers to mental health service user participation in health systems strengthening

These are broadly categorized as institutional, community and individual level barriers.

#### Institutional level barriers

The informants identified limited funding as one of the key barriers at institutional level, noting that funding for mental health activities has remained low despite an overall growth in funding to the health sector budget over the years. For example, it was reported that the resources used to form, train and develop mental health service user groups in the country came from the donor community with limited support from the government. At the district level, there are hardly any resources to support mental health service user groups.

*“As a country we are still struggling to fund mental health services and this affects the activities you are focusing on [mental health service user involvement”] (District Health Worker/Policy Maker II).*

Another barrier identified at this level was institutional stigma. It was noted that policy makers tend to hold a perception that people with mental illness are cognitively incompetent to effectively participate in policy making processes.

*“….really and first and foremost is stigma. People believe that once you have ever been diagnosed with a mental health disorder, you have no issues, no substance/important ideas to offer. I have seen it” (National Level Informant, Service User Organization I).*

*“When they are talking about HIV and AIDS they don’t say “those people”, but when they are talking about mental health [they refer to you as] they say “those people’’ and this means you are not fit to engage” (National Level Informant Service User Organization I).*

Some of the informants also identified patronage and abuse as a barrier to service user involvement; explained by the fact that most user organizations still struggle to outlive their founders. The situation is worsened by a tendency of abuse of powers by such founders. They cited instances of users penalized by having their entitlements withdrawn once they challenge the leadership.

*“…it is about me because I started this thing [user organization] or it is about me because am a leader here, and you fail to look beyond you an individual yet there are so many lives which are going to depend on this [user organization]” (National Level Informant, Service User Organization I).*

“…*because we talk when we have come to attend meetings… even though you are supposed to get transport refund, they don’t give it to you because you talk, you challenge, but for me I don’t stop talking even though they don’t give me” (National Level Key Informant Service User Organization I).*

#### Community level barriers

*Public stigma*: Mental health related stigma, which is partly attributed to limited sensitization was reported to prevail and identified as a key barrier to user involvement. Communities still stigmatize persons with mental illness, placing little value to their contribution. As a result, many of the people with mental illness were still reluctant to join service user organizations and their activities.

*“…people with mental illness are neglected and the community usually hates them because sometimes they are violent. They fail to come out openly to join most activities due to community stigma and this affects user group activities” (District Health Worker and Policy Maker I).*

Another factor identified to be a barrier to user involvement was poverty. As some of the service user group activities are strategic and expected to bear fruits in the future (such as advocacy), they were reported to have limited appeal to most members of the user groups who normally focus on immediate benefits.

*“Many of the group members left us once allowances for group members got finished. They want immediate things but not long term” (District Level Informant, Service User Organization I).*

#### Individual barriers

At individual level, informants identified lack of awareness and information as another key barrier. Most users were said to be unaware that it was their right to participate in health systems strengthening activities. This is worsened by limited information and lack of client empowerment, which makes many of the people within service user groups to grapple with self-stigma and hence limited participation in mental health service development activities.

*“You try to keep to yourself as much as possible so that other people do not get to know about your problem. And sometimes it is you who has the feeling that you are not able to contribute to group or community activities” (District Level Informant Service User Organization II).*

### Ways forward

The informants made a number of suggestions to improve service use involvement in mental health service development. These include raising awareness and building capacity of the service users, promoting activism to improve the plight of people with mental illness and their participation and promoting partnerships in the service user movement. They were cognizant of the fact that poverty limits the involvement of most mental health service users in mental health service development activities, and emphasized the importance of financial empowerment through income-generation as one possible strategy to overcome this barrier.

## Discussion

This is the first study to have carried out an in-depth assessment of the experience and barriers to engagement of mental health service users in health systems strengthening in Uganda. Research evidence is lacking in the country to support health reforms and program development [15, 25, 50]. This study provides some of the evidence. Similar to previous studies [3, 34, 35], this study reports low service user involvement in health systems strengthening. Informants reported that policy makers were not keen to integrate the views of service users in health systems development. The process seemed to be top-down and this was seen as a threat to sustainable improvement in health service delivery [1]. Despite this low level of involvement, informants were quite clear of the benefits of service user involvement in health systems strengthening. Some of the benefits included: improving the responsiveness of the health system to respect the actual needs of the users, reduced stigma and reducing the human resource gaps in the delivery system through task shifting with service users. These benefits are not unique to Uganda and have been mentioned in other studies in both LMICs [1–4, 10] and high-income countries [11–13, 16, 51, 52]. One of the glaring limitations to service user involvement is the lack of a clear strategy or a model to guide service user involvement in this process [4, 6]. The strategy and model envisaged needs to be clearly stated in legal and policy frameworks. Uganda has several laws and policies [e.g., the Uganda Ministry of Health Policy (2016), Uganda Ministry of Health Strategic, 2018–2022], however, these policies lack guidance on how service users could play an effective role in health systems strengthening. In health systems in the Western World, policy and legal frameworks exist to support service user involvement and collaboration [6, 10]. Uganda is currently at the stage of improving coverage and effectiveness of its PHC program, of which mental health is part. Such efforts need to be guided by legal and policy frameworks [1, 4].

In terms of research, our informants again reported low service user involvement in mental health research. Yet, user involvement improves not only the quality but also the impact and reach of research [6]. Findings indicated a few studies with an agenda to involve users. The majority of these studies were reported to be hospital-based and the concept of service user involvement became more tenuous as one moved to the district level. Informants described their participation in mental health research as tokenistic as they were not given the opportunity to give substantial input into the research process. Similar findings have been reported in related studies [1, 3, 4]. Addressing this challenge requires a deliberate effort to empower service users to demand for their rights to effectively participate in health research [53, 54]. It has been observed that many organizations that fund research today now ask researchers about user involvement as part of the application process [6]. For example, it has been reported that the National Institute of Health Research (NIHR) encourages researchers to involve service users in their work and plans for research and this is part of the standard application for funding [6]. Funding agencies that support mental health research should make service user involvement an essential requirement. The research work plans should clearly outline the activities where users would be involved as it is done in other research platforms/contexts such as the UK, where it is currently popular [6]. Also, ethical review boards should embed user involvement in mental health research as one of the key moral and ethical requirements for approving a study. The ethical boards in Uganda should have competencies and resources to monitor the work plans submitted by research groups.

In our study, the informants fought the notion of collective representation by organizations that were not specialized for mental health. Their key argument was that, though they had a mental illness, they did not identify themselves as being ‘disabled’ and, therefore, cannot be effectively represented by umbrella disability organizations in policy development and other activities that involve health systems strengthening. Furthermore, by incorporating mental health service user representation within umbrella organizations established for people with physical disability, there was concern that this would connote cognitive/intellectual disability and be used to deny them full or equal participation on issues concerning their plight as mental health service users [4, 55–57]. National frameworks should promote inclusion by mainstreaming user involvement in health systems strengthening. More advocacy is also needed here to support user groups to take on the policy makers to increase their visibility in such vital policy frameworks [4]. In addition, service user groups can join caregivers groups and form a common platform that aims at increasing their visibility in policy and program issues in the country [1, 4, 6]. National level user organizations can be at the forefront of policy engagement since they have more expertise and resources when compared to district based user organizations [3].

A number of barriers to user involvement in mental health research were also identified. Stigma seemed to be a major barrier to user involvement in mental health system strengthening and this has been acknowledged in related studies undertaken in low- and middle-income settings [1, 4, 55]. Self-stigma, as was evident in our study, makes users surrender to their plight and become less likely to challenge the status quo. For example, at the district level, users in this study reportedly feared to come out openly to declare that they had a mental illness and were also not keen to openly join service user groups and their activities. Several studies have documented how stigma towards service users is pervasive. The Uganda Ministry of Health should develop anti-stigma programs in their strategic plans. Investigators have advocated for a concerted effort between government and civil society agencies to avail resources and strategies that can ensure that users enjoy their full rights [4, 55–57]. The work plans for empowering service users can be integrated into other programs targeting conditions/diseases that also carry stigma (such as HIV/AIDS, cancer and tuberculosis) for cost efficiency and effective coordination [4]. Uganda has several programs on HIV/AIDS that have attracted a lot of resources from the donor community and this opportunity should be exploited. The approach should however be multi-level (from parish, sub-county, district and national) and multi-sectoral to draw meaningful resources for this endeavor. Uganda has recently been working on revision of mental health legislation (the Mental Health Bill, 2008). This needs to be scrutinized to ensure that it guarantees the rights of users and conforms with international legal frameworks [4, 54, 55].

Mental health leadership also seems to be a challenge that affects user groups. Our findings indicate that the user groups had internal struggles and lacked effective systems to work towards mental health service and system strengthening. The Uganda Ministry of Health, with support from donor agencies, should empower user groups on how they can deal with conflicts and strive towards fair and effective leadership. In our study, service user respondents had limited access to information, which may be exacerbated by the social exclusion of people with mental health conditions [30, 41]. Mental health awareness campaigns should be encouraged to promote mental health literacy.

In summary, there is a very strong moral case to support service user involvement in health systems strengthening [6, 58] in Uganda. In this study, mental health service users tended to be invisible, marginalized and not mobilized as a group. Structural barriers, including stigma and low funding, exacerbated the situation. Strategic steps need to be undertaken to develop the user movement in Uganda so that people with lived experience of mental health conditions can effectively engage in health systems strengthening activities. There is a possibility to integrate some of their activities in other government efforts in PHC. Legal and policy frameworks should be developed to support activities on-the-ground.

### Limitations and scope of the study

One limitation of the study was the small sample size, which limits the generalizability of our study findings. We sought to deal with this limitation through triangulation (we collected data from mental health workers/managers and users). Our findings can, however, be used for theoretical generalization [40, 42] with related studies undertaken in other contexts [1, 3, 4].

## Data Availability

Not applicable.

## Abbreviations

EMERALD: Emerging Mental Health Systems in Low- and Middle-Income Countries
LMIC: low- and middle-income country
MNS: mental, neurological and substance use
mhGAP: WHO’s Mental Health Gap Action Programme
MHCP: mental health care plan
NUDUP: National Union of Disabled Persons
PHC: primary health care
PRIME: Programme for Improving Mental Health Care
